# Positive association between different triglyceride glucose index-related indicators and psoriasis: evidence from NHANES

**DOI:** 10.3389/fimmu.2023.1325557

**Published:** 2023-12-20

**Authors:** Dawei Huang, Rui Ma, Xiaoyuan Zhong, Yuxiong Jiang, Jiajing Lu, Ying Li, Yuling Shi

**Affiliations:** ^1^ Department of Dermatology, Shanghai Skin Disease Hospital, Tongji University School of Medicine, Shanghai, China; ^2^ Institute of Psoriasis, Tongji University School of Medicine, Shanghai, China

**Keywords:** psoriasis, insulin resistance, TyG-BMI, TyG-WC, TyG-WHtR

## Abstract

**Background:**

Psoriasis is a chronic inflammatory skin disease with effects that extend beyond the skin. Insulin resistance (IR) has been associated with psoriasis, but it remains unclear how indicators related to the triglyceride glucose (TyG) index, which were associate with IR, are associated with the condition.

**Objective:**

The purpose of this study was to investigate the association between psoriasis and three TyG-related indicators: triglyceride glucose-body mass index (TyG-BMI), triglyceride glucose-waist to height ratio (TyG-WHtR), and triglyceride glucose-waist circumference (TyG-WC).

**Methods:**

Data from adults aged 20 to 80 years in the National Health and Nutrition Examination Survey (NHANES) from 2003 to 2006 and 2009 to 2014 were utilized. Institutional Review Board approval and documented written consent was obtained from participants by NHANES (Protocol #2005–06). The patients were divided into three groups based on TyG-BMI, TyG-WC, and TyG-WHtR: Q1 (1st quintile), Q2 (2nd-3rd quintiles), and Q3 (4th-5th quintiles). Differences between the groups were further explored. Multivariate logistic regressions were used to investigate the correlation between these three indicators and psoriasis, with results expressed as odds ratios (OR) and 95% confidence intervals (CI). Subgroup analysis and supplementary analysis was further conducted to explore potential influencing factors.

**Results:**

The study included 9,291 participants, of which 260 had psoriasis. Compared Q2 and Q3 of TyG-BMI, TyG-WC, and TyG-WHtR to Q1, there were significantly associate with psoriasis. Among the three indicators, TyG-WC consistently had the highest OR values in Models 1 and 2 (Model 1: Q3 OR (95% CI) = 2.155 (1.442-3.220); Model 2: Q3 OR (95% CI) = 2.029 (1.341-3.069)). While in Model 3, the TyG-BMI shows more significant relationship with psoriasis (Model 3 of TyG-BMI: Q3 OR (95% CI) = 1.948 (1.300-3.000)). Similar results were observed in the majority of subgroups and in supplementary analysis.

**Conclusion:**

This study identified a stable and strong positive association between TyG-related indicators (TyG-BMI, TyG-WC, and TyG-WHtR) and psoriasis. This association persisted even after adjusting for multiple factors. It is suggested that high IR is significantly associated with psoriasis.

## 1 Introduction

Psoriasis is a chronic inflammatory skin disorder that affects approximately 2-3% of the global population (1). It is characterized by red, scaly patches on the skin, which can cause significant physical and psychological distress for those affected. The exact cause of psoriasis is not fully understood, but it is believed to be a multifactorial disease with both genetic and environmental factors playing a role (2).

Psoriasis has effects on the body that extend beyond the skin. For instance, individuals with psoriasis have a heightened risk of developing metabolic syndrome and cardiovascular disease (3). Insulin resistance (IR) may play a pivotal role in this association. In patients with psoriasis and metabolic syndrome, inflammatory factors remain consistently elevated, further exacerbating IR and potentially serving as a mechanism for the development of psoriasis with metabolic syndrome (4). Thus, it is crucial to comprehend the correlation between IR and psoriasis.

The traditional evaluation of IR is time-consuming and laborious, typically utilizing the hyperinsulinemic-euglycemic clamp (HIEC) as the gold standard. However, new tools for evaluating IR have been developed in recent years, which greatly facilitate their clinical application, such as the homeostasis model assessment of insulin resistance (HOMA-IR) and the Quantitative Insulin Sensitivity Check Index (QUICKI). One such tool is the triglyceride glucose (TyG) index, which is receiving attention due to its strong correlation with HIEC (5). Building upon the TyG index, several new indicators for assessing IR have been developed to provide a more accurate evaluation of its severity. These include the triglyceride glucose-body mass index (TyG-BMI) (6), the triglyceride glucose-waist to height ratio (TyG-WHtR) (7), and the triglyceride glucose-waist circumference (TyG-WC) (8), all of which have been reported as reliable alternative markers of IR.

Given the need for more reliable evidence regarding the association between insulin resistance and psoriasis in patients, the aim of this study was to assess this association using various indicators related to TyG, and to provide valuable insights into the relationship between insulin resistance and psoriasis.

## 2 Methods

### 2.1 Study design

**Figure 1:**
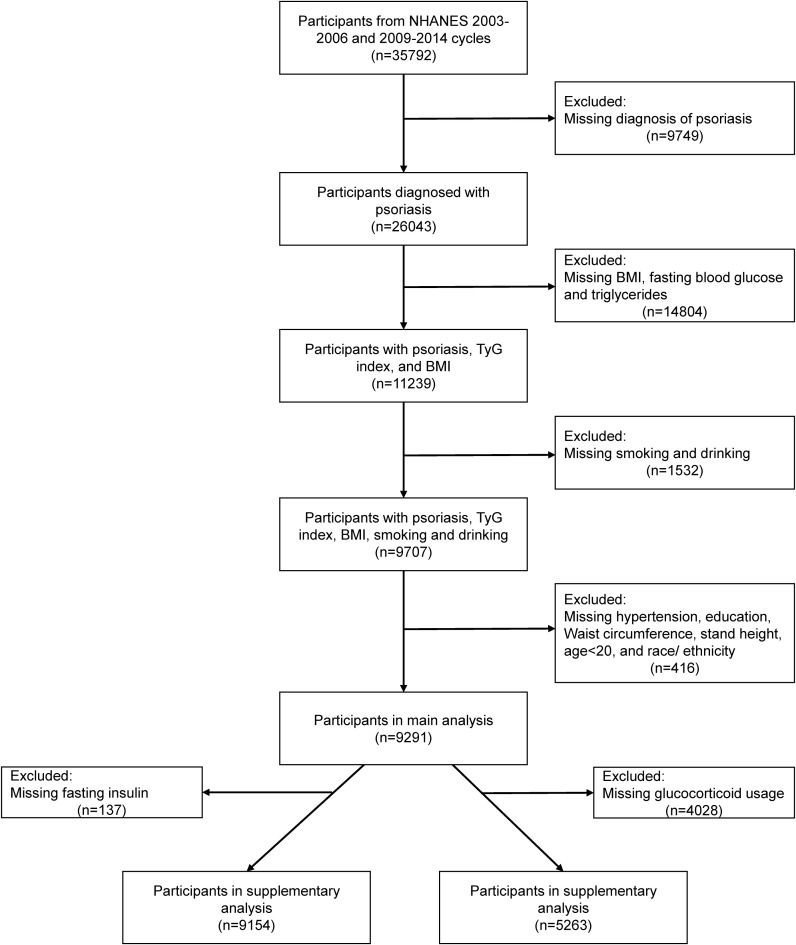
Flow chart of participant selection. BMI, body mass index; NHANES, National Health and Nutrition Examination Survey; TyG index, triglyceride glucose index.

The research design of this study was based on a cross-sectional study design, and the data analyzed were obtained from the National Health and Nutrition Examination Survey (NHANES). NHANES is a large, nationwide survey that assesses the health status of citizens by collecting demographic, health, economic, and various other types of data (9). Institutional Review Board approval and documented written consent was obtained from participants by NHANES (Protocol #2005–06). The survey was conducted with the approval of the National Center for Health Statistics Ethics Review Committee, and all participants provided written informed consent. The exclusion criteria of main analysis included: 1) without psoriasis information; 2) age <20 years old (psoriasis data on participants <20 years of age are missing in 2003–2004, and 2005–2006 cycles); 3) missing data on fasting blood glucose, triglycerides, BMI, smoking status, drinking status, hypertension, diabetes, waist circumference, stand height and race/ethnicity. Ultimately, 9291 participants were included for analysis (**Figure 1**).

### 2.2 Data collection

All analysis data were directly extracted from the database and included variables such as age, gender, education, BMI, height, waist circumference, smoking history, alcohol history, hypertension history, diabetes history, and psoriasis history. We extracted data for a total of five cycle years (a span of 10 years), specifically from 2003 to 2006 and from 2009 to 2014. Additionally, blood samples were collected from all participants in the early morning to analyze fasting blood glucose, fasting insulin and triglyceride levels. Smoking and drinking status were categorized as “Never,” “Current,” or “Past.” The educational level was divided into two categories: high school and below, and college and above, as stated in the questionnaire. BMI, waist circumference, and height measurements were conducted at a mobile examination center. Glucocorticoids usage was defined if the participants responded affirmatively to the question, “Ever taken prednisone or cortisone daily” (10). Psoriasis information was also obtained through self-reports on a health questionnaire, where participants were asked, “Have you ever been told by a health care provider that you had psoriasis?” A response of “yes” indicated a case of “psoriasis.” (11). Hypertension was diagnosed by systolic pressure/diastolic pressure ≥ 140/90 mmHg, or self-reported physician diagnosis of hypertension, or self-reported use of hypertension medication. Diabetes was diagnosed by glycosylated hemoglobin ≥ 6.5%, or self-reported physician diagnosis of diabetes, or self-reported use of insulin (12). The waist-to-height ratio (WHtR) was defined as the waist circumference (WC) divided by standing height.

Additionally, the calculation formula for the TyG index was as follows: TyG = Ln [fasting triglyceride (mg/dL) × fasting blood glucose (mg/dL)/2] (13). TyG-BMI was calculated as TyG index multiplied by BMI (6); Tyg-WC was calculated as TyG multiplied by WC (14); TyG-WHtR was calculated as TyG multiplied by WHtR (7); HOMA-IR = (fasting insulin (mU/L) × fasting blood glucose (mg/dL)/18)/22.5 (15); QUICKI = 1/[log (fasting insulin (μU/mL)) +log (fasting blood glucose (mg/dL))] (16).

### 2.3 Statistical analysis

Continuous variables described by a normal distribution are expressed as mean ± standard deviation (mean± sd). Non-normally distributed data is expressed as the median (interquartile range [IQR]). Categorical variables are expressed as numbers (n) and percentages (%). To further examine the association between TyG-related indicators and psoriasis, we divided all patients into three equal groups based on quintile of TyG-BMI, TyG-WC, and TyG-WHtR. The 1st quintile was defined as Q1. And the 2nd-3rd, and 4th-5th quintiles was regarded as a single group, respectively defined as Q2 and Q3. Although sampling weights are usually taken into consideration to produce representative and unbiased statistics during the analysis of a complex survey, it could also reduce the precision of the estimates. Besides, sampling weights could even bring some extent introduces over-adjustment bias (17–19). Thus, we performed analysis without incorporating sampling weights, consistent to some previous research using NHANES data (17–19). We used the Chi-square test or the Kruskal-Wallis H test to compare in different groups. Based on previous literature, covariates included age, gender, education level, smoking status, drinking status, hypertension, diabetes, and race/ethnicity (20–22). To further investigate the association, we established three models: Model 1 did not include any covariates, Model 2 only adjusted for age and gender, and Model 3 included all covariates for analysis. Multivariate logistic regression was used to assess the association between different TyG-related indicators and psoriasis in these three models, with odds ratios (OR) and 95% confidence intervals (CI) used to indicate the strength of the relationship. Besides, we also used restricted cubic splines to explore the non-linear dose-response relationship between TyG-related indicators and psoriasis, selecting knots that minimized the akaike information criterion (23). Additionally, we conducted several subgroup and interaction analyses to identify potential contributing factors. Given the high values of TyG-BMI and TyG-WC, they were transformed by natural logarithm (Ln-transformed) in subgroup analysis, which did not change the original trend of the data. We conducted a supplementary analysis, calculating the HOMA-IR and QUICKI indices of the patients and determining the OR values under different models. Due to generally right-skewed distribution of all TyG-related indicators, HOMA-IR, and QUICKI, thus these variables were converted into natural logarithms during the supplementary analysis. Because the use of corticosteroids can significantly impact insulin resistance, we included corticosteroids as a covariate in Model 3 to further analyze the relationship of TyG-related indicators in psoriasis in supplementary analysis. R 4.2.1 was used for all statistical analyses. All tests were two-sided, and P value < 0.05 was considered statistically significant.

## 3 Results

### 3.1 Characteristics of the participants

Among the 9291 participants in our main analysis, we grouped these participants based on whether or not they had psoriasis and then compared their baseline conditions. A total of 260 patients with psoriasis were identified. The psoriasis patients were found to have a higher average age, greater BMI, larger waist circumference, and higher WHtR compared to the non-psoriasis participants. Additionally, a higher proportion of psoriasis patients reported being smokers. Furthermore, psoriasis patients had a higher incidence of hypertension and higher triglyceride levels. All TyG-related indicators were also found to be significantly higher in psoriasis patients compared to non-psoriasis participants (**Table 1**). These results suggest that patients with psoriasis may have more obvious insulin resistance. Then, we divided all patients into Q1, Q2, and Q3 groups based on their TyG-BMI, TyG-WC, and TyG-WHtR, and then compared the differences among these groups. Our analysis revealed that regardless of which indicator was used for classification, Q3 patients consistently exhibited a higher prevalence of psoriasis, older age, higher BMI, waist circumference, and WHtR. Additionally, they had a lower educational level, were more likely to smoke and consume alcohol, had a higher prevalence of hypertension and diabetes, and exhibited higher levels of fasting blood glucose and triglycerides compared to Q1 and Q2 patients (**Table 2**).

**Table 1:** Comparison of characteristics of psoriasis patients and non-psoriasis participants.

Characteristics	Total (n=9291)	Non-psoriasis (n=9031)	Psoriasis (n=260)	P value
**Gender, n (%)**				0.596
Female	4745 (51.1%)	4608 (51.0%)	137 (52.7%)	
Male	4546 (48.9%)	4423 (49.0%)	123 (47.3%)	
**Age (year), mean ± sd**	46.2 ± 16.9	46.1 ± 16.9	49.2 ± 16.1	0.003
**BMI (kg/m^2^), mean ± sd**	28.9 ± 6.8	28.9 ± 6.8	30.5 ± 7.4	< 0.001
**Waistline (cm), mean ± sd**	98.7 ± 16.3	98.6 ± 16.3	103.0 ± 17.1	< 0.001
**Stand height (cm), mean ± sd**	168.0 ± 10.0	168.0 ± 10.0	167.5 ± 9.6	0.436
**WHtR, mean ± sd**	0.59 ± 0.10	0.59 ± 0.10	0.62 ± 0.10	< 0.001
**Education level, n (%)**				0.139
High school and below	4314 (46.4%)	4205 (46.6%)	109 (41.9%)	
College and above	4977 (53.6%)	4826 (53.4%)	151 (58.1%)	
**Race/ethnicity, n (%)**				< 0.001
Mexican American	1511 (16.3%)	1488 (16.5%)	23 (8.8%)	
White	4282 (46.1%)	4122 (45.6%)	160 (61.5%)	
Black	1841 (19.8%)	1807 (20.0%)	34 (13.1%)	
Other Hispanic	789 (8.5%)	770 (8.5%)	19 (7.3%)	
Other race	868 (9.3%)	844 (9.3%)	24 (9.2%)	
**Smoking, n (%)**				0.020
Never	5143 (55.4%)	5017 (55.6%)	126 (48.5%)	
Current	2034 (21.9%)	1977 (21.9%)	57 (21.9%)	
Past	2114 (22.8%)	2037 (22.6%)	77 (29.6%)	
**Drinking, n (%)**				0.348
Never	1256 (13.5%)	1226 (13.6%)	30 (11.5%)	
Current	6478 (69.7%)	6299 (69.7%)	179 (68.8%)	
Past	1557 (16.8%)	1506 (16.7%)	51 (19.6%)	
**Diabetes, n (%)**				0.456
No	8110 (87.3%)	7887 (87.3%)	223 (85.8%)	
Yes	1181 (12.7%)	1144 (12.7%)	37 (14.2%)	
**Hypertension, n (%)**				< 0.001
No	5801 (62.4%)	5674 (62.8%)	127 (48.8%)	
Yes	3490 (37.6%)	3357 (37.2%)	133 (51.2%)	
**Fasting blood glucose (mg/dL), mean ± sd**	105.3 ± 32.4	105.12 ± 32.4	108.4 ± 33.0	0.112
**Triglyceride (mg/dL), mean ± sd**	132.7 ± 118.4	132.3 ± 117.6	147.0 ± 141.5	0.048
**TyG-BMI, mean ± sd**	251.1 ± 67.1	250.6 ± 66.8	268.4 ± 73.7	< 0.001
**TyG-WC, mean ± sd**	856.0 ± 177.1	854.6 ± 176.6	905.1 ± 187.8	< 0.001
**TyG-WHtR, mean ± sd**	5.1 ± 1.1	5.1 ± 1.1	5.4 ± 1.1	< 0.001

n, number; BMI, body mass index; WHtR, waist to height ratio; sd, standard deviation; TyG-BMI, triglyceride glucose-body mass index; TyG-WC, triglyceride glucose-waist circumference; TyG-WHtR, triglyceride glucose-waist to height ratio.

**Table 2:** Differences among groups were compared according to TyG-related indicators.

Characteristics	TyG-BMI				TyG-WC				TyG-WHtR			
	Q1	Q2	Q3	P value	Q1	Q2	Q3	P value	Q1	Q2	Q3	P value
**Number**	1857	3715	3719		1858	3715	3718		1847	3711	3733	
**Psoriasis, n (%)**	31 (1.7%)	104 (2.8%)	125 (3.4%)	0.001	30 (1.6%)	103 (2.8%)	127 (3.4%)	< 0.001	30 (1.6%)	103 (2.8%)	127 (3.4%)	< 0.001
**Female, n (%)**	1078 (58.1%)	1719 (46.3%)	1948 (52.4%)	< 0.001	1202 (64.7%)	1887 (50.8%)	1656 (44.5%)	< 0.001	962 (52.1%)	1735 (46.8%)	2048 (54.9%)	< 0.001
**Age (year), median (IQR)**	37 (25, 52)	46 (33, 60)	47 (35, 60)	< 0.001	34 (25, 47)	46 (33, 59)	49 (36, 62)	< 0.001	33 (25, 46)	46 (33, 59)	50 (37, 63)	< 0.001
**BMI (kg/m^2^), median (IQR)**	21.5 (20.1, 22.7)	26.47 (24.9, 28.0)	33.6 (30.9, 37.9)	< 0.001	22.0 (20.2, 23.8)	26.6 (24.5, 29.0)	33.0 (29.8, 37.7)	< 0.001	22.0 (20.2, 23.7)	26.6 (24.5, 28.9)	33.1 (29.8, 37.7)	< 0.001
**Waistline (cm), median (IQR)**	79.5 (75.0, 84.2)	93.6 (88.5, 99.0)	110.6 (103.7, 119.9)	< 0.001	79.1 (75.0., 82.9)	93.6 (89.1, 98.2)	110.9 (104.6, 119.9)	< 0.001	79.5 (75.0, 84.1)	93.8 (88.5, 99.3)	110.5 (103.1, 119.8)	< 0.001
**WHtR, median (IQR)**	0.47 (0.45, 0.50)	0.56 (0.53, 0.59)	0.66 (0.62, 0.72)	< 0.001	0.47 (0.45, 0.5)	0.56 (0.53, 0.59)	0.66 (0.61, 0.72)	< 0.001	0.47 (0.44, 0.50)	0.56 (0.53, 0.58)	0.66 (0.62, 0.72)	< 0.001
**Education level, n (%)**				< 0.001				< 0.001				< 0.001
High school and below	708 (38.1%)	1696 (45.7%)	1910 (51.4%)		671 (36.1%)	1728 (46.5%)	1915 (51.5%)		660 (35.7%)	1647 (44.4%)	2007 (53.8%)	
College and above	1149 (61.9%)	2019 (54.3%)	1809 (48.6%)		1187 (63.9%)	1987 (53.5%)	1803 (48.5%)		1187 (64.3%)	2064 (55.6%)	1726 (46.2%)	
**Race/ethnicity, n (%)**				< 0.001				< 0.001				< 0.001
Mexican American	186 (10.0%)	605 (16.3%)	720 (19.4%)		208 (11.2%)	602 (16.2%)	701 (18.9%)		166 (9.0%)	557 (15.0%)	788 (21.1%)	
White	923 (49.7%)	1680 (45.2%)	1679 (45.1%)		847 (45.6%)	1617 (43.5%)	1818 (48.9%)		901 (48.8%)	1674 (45.1%)	1707 (45.7%)	
Black	320 (17.2%)	711 (19.1%)	810 (21.8%)		374 (20.1%)	768 (20.7%)	699 (18.8%)		407 (22.0%)	772 (20.8%)	662 (17.7%)	
Other Hispanic	117 (6.3%)	342 (9.2%)	330 (8.9%)		137 (7.4%)	341 (9.2%)	311 (8.4%)		112 (6.1%)	316 (8.5%)	361 (9.7%)	
Other race	311 (16.7%)	377 (10.1%)	180 (4.8%)		292 (15.7%)	387 (10.4%)	189 (5.1%)		261 (14.1%)	392 (10.6%)	215 (5.8%)	
**Smoking, n (%)**				< 0.001				< 0.001				< 0.001
Never	1081 (58.2%)	2060 (55.5%)	2002 (53.8%)		1163 (62.6%)	2106 (56.7%)	1874 (50.4%)		1102 (59.7%)	2075 (55.9%)	1966 (52.7%)	
Current	484 (26.1%)	753 (20.3%)	797 (21.4%)		424 (22.8%)	806 (21.7%)	804 (21.6%)		485 (26.3%)	769 (20.7%)	780 (20.9%)	
Past	292 (15.7%)	902 (24.3%)	920 (24.7%)		271 (14.6%)	803 (21.6%)	1040 (28%)		260 (14.1%)	867 (23.4%)	987 (26.4%)	
**Drinking, n (%)**				< 0.001				< 0.001				< 0.001
Never	262 (14.1%)	468 (12.6%)	526 (14.1%)		260 (14.0%)	508 (13.7%)	488 (13.1%)		231 (12.5%)	457 (12.3%)	568 (15.2%)	
Current	1380 (74.3%)	2653 (71.4%)	2445 (65.7%)		1407 (75.7%)	2625 (70.7%)	2446 (65.8%)		1446 (78.3%)	2669 (71.9%)	2363 (63.3%)	
Past	215 (11.6%)	594 (16.0%)	748 (20.1%)		191 (10.3%)	582 (15.7%)	784 (21.1%)		170 (9.2%)	585 (15.8%)	802 (21.5%)	
**Diabetes, n (%)**				< 0.001				< 0.001				< 0.001
No	1812 (97.6%)	3397 (91.4%)	2901 (78.0%)		1838 (98.9%)	3425 (92.2%)	2847 (76.6%)		1823 (98.7%)	3460 (93.2%)	2827 (75.7%)	
Yes	45 (2.4%)	318 (8.6%)	818 (22.0%)		20 (1.1%)	290 (7.8%)	871 (23.4%)		24 (1.3%)	251 (6.8%)	906 (24.3%)	
**Hypertension, n (%)**				< 0.001				< 0.001				< 0.001
No	1499 (80.7%)	2451 (66.0%)	1851 (49.8%)		1572 (84.6%)	2453 (66.0%)	1776 (47.8%)		1570 (85.0%)	2466 (66.5%)	1765 (47.3%)	
Yes	358 (19.3%)	1264 (34.0%)	1868 (50.2%)		286 (15.4%)	1262 (34.0%)	1942 (52.2%)		277 (15.0%)	1245 (33.5%)	1968 (52.7%)	
**Fasting blood glucose (mg/dL), median (IQR)**	92.0 (86.0, 98.0)	97.0 (90.3, 104.4)	103.0 (95.4, 117.0)	< 0.001	91.0 (86.0, 97.0)	97.0 (90.9, 104.0)	105.0 (96.0, 118.0)	< 0.001	91.2 (86.0, 97.0)	97.0 (91.0, 104.0)	104.0 (96.0, 119.0)	< 0.001
**Triglyceride (mg/dL), median (IQR)**	69.0 (53.0, 91.0)	100.0 (74.0, 137.0)	144.0 (100.0, 208.0)	< 0.001	65.5 (51.0, 86.0)	98.0 (73.0, 130.0)	150.0 (107.0, 216.0)	< 0.001	65.0 (51.0, 85.0)	98.0 (74.0, 130.0)	150.0 (107.0, 215.0)	< 0.001

n, number; BMI, body mass index; WHtR, waist to height ratio; IQR, interquartile range; TyG-BMI, triglyceride glucose-body mass index; TyG-WC, triglyceride glucose-waist circumference; TyG-WHtR, triglyceride glucose-waist to height ratio.

### 3.2 Associations between TyG-related indicators and psoriasis

**Figure 2:**
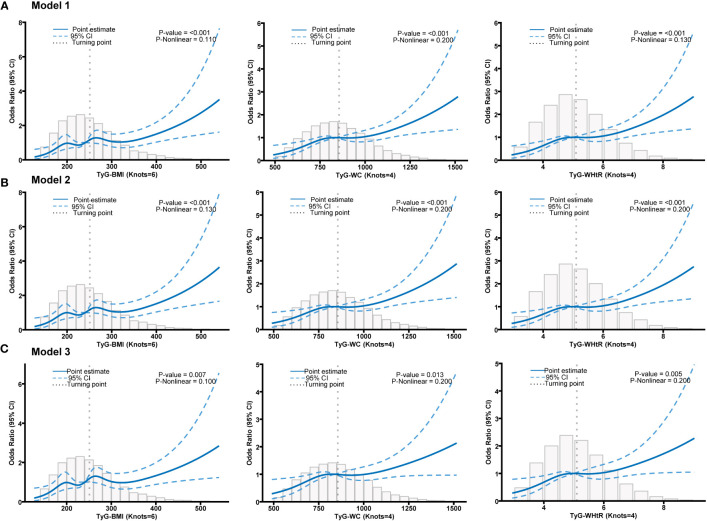
**(A)** Restricted cubic spline fitting for the association between different TyG-related indicators with psoriasis in model 1. **(B)** Restricted cubic spline fitting for the association between different TyG-related indicators with psoriasis in model 2. **(C)** Restricted cubic spline fitting for the association between different TyG-related indicators with psoriasis in model 3.

**Table 3** presents the OR and their corresponding 95% confidence intervals, which depict the association between various TyG-related indicators and psoriasis. These associations were analyzed using three different models. The results revealed an independent positive association between TyG-BMI, TyG-WC, and TyG-WHtR and psoriasis in various models. Additionally, Q2 and Q3 of all indicators exhibited a significant positive relationship with psoriasis in comparison to Q1 (**Table 3**). This suggests that higher TyG-related indicators serve as independent risk factors for psoriasis. Among the three indicators, TyG-WC displayed a greater OR value in Models 1 and 2 (Model 1, Q3 OR (95% CI) = 2.155 (1.442-3.220); Model 2, Q3 OR (95% CI) = 2.029 (1.341-3.069)). While in Model 3, the TyG-BMI shows more significant relationship with psoriasis (Model 3 of TyG-BMI: Q3 OR (95% CI) = 1.948 (1.300-3.000)). The restricted cubic spline analysis (**Figure 2**) also demonstrated a positive linear correlation between TyG-BMI, TyG-WC, and TyG-WHtR with psoriasis in all models (P-value < 0.05, P-nonlinear > 0.05).

**Table 3:** Multivariate regression analysis of TyG-related indicators with psoriasis.

Exposures	Model 1 Crude OR (95% CI)	Model 2 Adjusted OR (95% CI)	Model 3 Adjusted OR (95% CI)
TyG-BMI			
Q1	Ref.	Ref.	Ref.
Q2	1.696 (1.132-2.543)	1.610 (1.070-2.424)	1.679 (1.126-2.574)
Q3	2.049 (1.377-3.048)	1.933 (1.295-2.886)	1.948 (1.300-3.000)
TyG-WC			
Q1	Ref.	Ref.	Ref.
Q2	1.738 (1.153-2.619)	1.650 (1.087-2.506)	1.683 (1.121-2.599)
Q3	2.155 (1.442-3.220)	2.029 (1.341-3.069)	1.928 (1.269-3.011)
TyG-WHtR			
Q1	Ref.	Ref.	Ref.
Q2	1.729 (1.147-2.606)	1.612 (1.062-2.448)	1.638 (1.093-2.527)
Q3	2.133 (1.427-3.188)	1.930 (1.275-2.920)	1.906 (1.257-2.971)

Model 1 adjust for: None; Model 2 adjust for: age, gender; Model 3 adjust for: age, gender, education, smoking, drinking, hypertension, diabetes, and race/ethnicity; Abbreviation: OR, odds ratios; 95% CI, 95% confidence intervals; TyG-BMI, triglyceride glucose-body mass index; TyG-WC, triglyceride glucose-waist circumference; TyG-WHtR, triglyceride glucose-waist to height ratio; Ref., reference.

**Figure 3:**
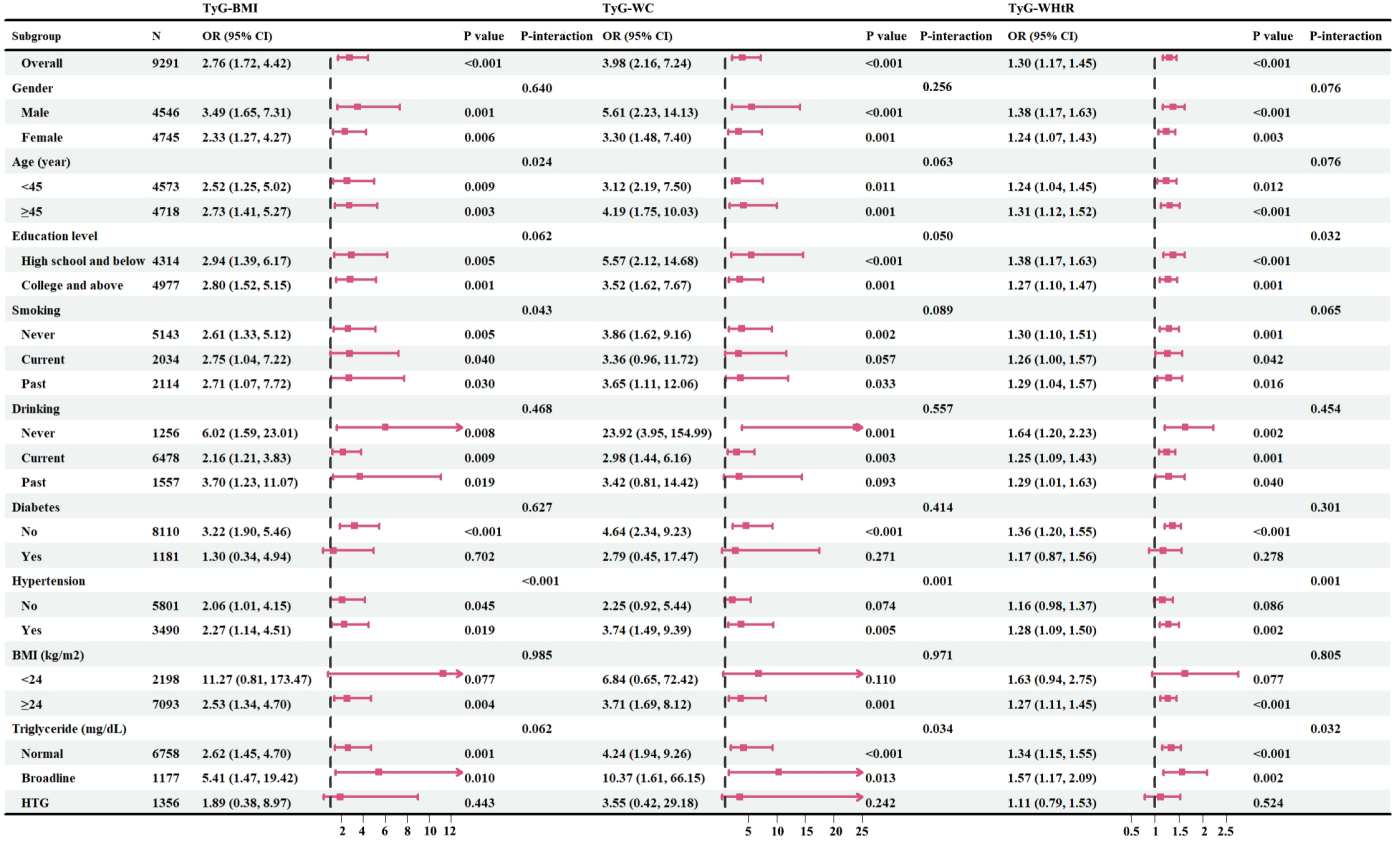
Forest maps of subgroup analysis. Fasting plasma triglyceride concentrations may be categorized as normal (<150 mg/dL), borderline (150-199 mg/dL), high triglyceride (HTG>200 mg/dL); n, number; OR, odds ratios; 95% CI, 95% confidence intervals; BMI, body mass index; TyG-BMI, triglyceride glucose-body mass index; TyG-WC, triglyceride glucose-waist circumference; TyG-WHtR, triglyceride glucose-waist to height ratio; HTG, high triglyceride.

Subsequently, we conducted a subgroup analysis to further explore the relationship between TyG-related indicators and psoriasis (**Figure 3**). In the vast majority of stratified populations, the relationship between TyG-related indicators and psoriasis was similar, as no significant interaction was observed (P for interaction >0.05). However, for patients with hypertension and hypertriglyceridemia, higher TyG-WC and TyG-WHtR did not indicate a higher risk of psoriasis. On the other hand, TyG-BMI still showed a positive relationship trend as it had no interaction with hypertension and hypertriglyceridemia (P for interaction >0.05). What’s more, subgroup analysis results suggest that even if a patient does not have diabetes, an elevated TyG-related index still represents a higher risk of psoriasis.

### 3.3 Supplementary analysis

To further elucidate the relationship between IR and psoriasis, we conducted a comparison of TyG-related indicators with HOMA-IR and QUICKI. The findings revealed that TyG-related indices exhibited a more significant relationship with psoriasis compared to traditional IR evaluation indices such as HOMA-IR and QUICKI (**Supplementary Table S1**).

The use of glucocorticoids can significantly impact insulin resistance. Therefore, we included the use of glucocorticoids as a covariate in Model 3 to further elucidate the roles of IR in psoriasis. However, due to the limited availability of data on the use of glucocorticoids in the NHANES database (2005-2006, 2009-2010, and 2013-2014), we will present the analysis of glucocorticoids as a supplementary analysis. The results demonstrated that even after adjusting for all covariates, including glucocorticoid usage, all TyG-related indicators remained positively associated with psoriasis (**Supplementary Table S2**).

## 4 Discussion

Psoriasis and IR are both significant health issues and they have a relationship with each other. The etiology of these two diseases is multifactorial, however, they do share certain similarities. One common triggering factor in both diseases is inflammation (24). For instance, thiazolidinediones have been shown to effectively improve insulin resistance and also contribute to plaque regression in patients with psoriasis (25). In addition, methotrexate can also have a pharmacological effect by directly acting on PPAR-gamma (26). Given that methotrexate is an important therapeutic drug for psoriasis, this further underscores the significant role of insulin resistance in the condition. Therefore, it is necessary to explore the correlation between IR and psoriasis.

This large, nationally representative, cross-sectional study evaluates the association between TyG-related indicators and psoriasis. Our study reveals a positive association between high TyG-BMI, TyG-WC, and TyG-WHtR, and psoriasis in adult U.S. citizens. This positive association remains stable even after adjusting for age, gender, educational experience, smoking, drinking, hypertension, and diabetes. Additionally, further subgroup analysis demonstrates that this positive relationship still persists in most subgroups and supplementary analysis. To the best of our knowledge, this study is the first to explore the relationship between TyG-related indicators and psoriasis.

The TyG index and its associated indicators have recently been extensively explored in cardiovascular disease (27, 28). Also, anthropometric data, such as BMI, WC, and WHtR, are accepted as predictors of obesity and IR. Recently, novel TyG-related indicators of the TyG index combined with anthropometric data have been derived, namely TyG-BMI, TyG-WC, and TyG-WHtR. Therefore, combining anthropometric and metabolic indices may be more reliable than TyG alone (29). A recent study confirmed that TyG-WHtR is more helpful than TyG in identifying high-risk prediabetes (30). In a large retrospective study with 214493 participants, it was found that the TyG-BMI were significantly associated with prehypertension (31). Additionally, TyG-BMI, TyG-WC, and TyG-WHtR had higher predictive performance for non-alcoholic fatty liver disease in the general population (32). Therefore, in this study, TyG-BMI, TyG-WC, and TyG-WHtR were used to determine the correlation between IR and psoriasis.

Although the exact causal relationship between IR and psoriasis has not been fully established, observational studies have shown that waist circumference has the strongest association with psoriasis, followed by high-density lipoprotein and triglyceride levels. Additionally, patients with persistent metabolic syndrome have a higher risk of developing psoriasis (33). Our own study supports these findings. However, the precise mechanism underlying this relationship is not fully understood. Although there are two-sample Mendelian randomization studies from a genetic perspective, metabolic syndrome, WC, and hypertension are risk factors for the development of psoriasis (34). One compelling hypothesis is that increased levels of inflammatory markers in individuals with IR may lead to a heightened risk of psoriasis. Both IR and psoriasis involve various inflammatory pathways, such as the Th17-mediated inflammatory pathway (35). Many similar cytokines contribute to the pathogenesis of both conditions. Furthermore, insulin resistance may contribute to the development of psoriatic metabolic disorders and skin inflammation by reducing the levels of anti-inflammatory adipokines, for example adiponectin (36, 37). Genetics also play a role in the correlation between psoriasis and metabolic syndrome. There are common susceptibility genes and genetic loci shared between the two conditions. For example, mutations in the *IL-12B*, *IL-23R*, and *IL-23A* genes are associated with both susceptibility to psoriasis and an increased risk of developing diabetes (38). Another genome-wide association study demonstrated that genes related to dyslipidemia, hypertension, and cardiovascular disease are associated with an increased risk of psoriasis (39). These findings collectively suggest a significant correlation between insulin resistance and psoriasis.

Our study has significant advantages. Firstly, we utilized a large nationwide sample size and implemented appropriate covariate adjustment, thereby enhancing the reliability of our findings. Secondly, our study comprehensively assessed the individual impact of TyG-related indicators on psoriasis risk, addressing a gap in previous research. Lastly, the availability of TyG-related indicators for assessing insulin resistance is based on routine clinical care, eliminating the need for specific insulin testing or HIEC, thus making it a readily accessible and cost-effective biomarker with potential clinical applications.

However, there are a few limitations worth noting in our study. Firstly, the cross-sectional study design restricts our ability to establish a causal relationship between insulin resistance and psoriasis. Secondly, the selection of psoriasis cases was based on self-reported questionnaires, which introduces the potential for recall bias. Finally, despite adjusting for some confounders, there may still be residual or unmeasured confounders affecting our results. Therefore, future longitudinal prospective studies with comprehensive data collection are crucial to confirm the association between TyG-BMI, TyG-WC, and TyG-WHtR and the risk of psoriasis.

## 5 Conclusions

In a nationally representative sample of U.S. adults, a stable and strong positive association was observed between TyG-related measures, specifically TyG-BMI, TyG-WC, and TyG-WHtR, and psoriasis. This association persisted even after adjusting for multiple factors. However, future high quality cohort studies should aim to verify this relationship in a wider population.

## Data availability statement

The original contributions presented in the study are included in the article/**Supplementary Material**. Further inquiries can be directed to the corresponding authors.

## Ethics statement

The studies involving humans were approved by Ethics Review Committee of the National Center for Health Statistics. The studies were conducted in accordance with the local legislation and institutional requirements. The participants provided their written informed consent to participate in this study.

## Author contributions

DH: Writing – original draft. RM: Data curation, Writing – original draft. XZ: Methodology, Resources, Writing – original draft. YJ: Methodology, Writing – original draft. JL: Funding acquisition, Writing – review & editing. YL: Writing – review & editing. YS: Funding acquisition, Writing – review & editing.

## References

[1] Griffiths C Armstrong AW Gudjonsson JE Barker J. Psoriasis. Lancet (2021) 397:1301–15. doi: 10.1016/S0140-6736(20)32549-6 33812489

[2] Lé AM Torres T. New topical therapies for psoriasis. Am J Clin Dermatol (2022) 23:13–24. doi: 10.1007/s40257-021-00649-w 34705167

[3] Kamiya K Kishimoto M Sugai J Komine M Ohtsuki M. Risk factors for the development of psoriasis. Int J Mol Sci (2019) 20(18):4347. doi: 10.3390/ijms20184347 31491865 PMC6769762

[4] Liu L Cai XC Sun XY Zhou YQ Jin MZ Wang J. Global prevalence of metabolic syndrome in patients with psoriasis in the past two decades: current evidence. J Eur Acad Dermatol Venereol (2022) 36:1969–79. doi: 10.1111/jdv.18296 35666614

[5] Nabipoorashrafi SA Seyedi SA Rabizadeh S Ebrahimi M Ranjbar SA Reyhan SK. The accuracy of triglyceride-glucose (TyG) index for the screening of metabolic syndrome in adults: A systematic review and meta-analysis. Nutr Metab Cardiovasc Dis (2022) 32:2677–88. doi: 10.1016/j.numecd.2022.07.024 36336547

[6] Er LK Wu S Chou HH Hsu LA Teng MS Sun YC. Triglyceride glucose-body mass index is a simple and clinically useful surrogate marker for insulin resistance in nondiabetic individuals. PloS One (2016) 11:e149731. doi: 10.1371/journal.pone.0149731 PMC4773118 26930652

[7] Mirr M Braszak-Cymerman A Ludziejewska A Kręgielska-Narożna M Bogdański P Bryl W. Serum asprosin correlates with indirect insulin resistance indices. Biomedicines (2023) 11(6):1568. doi: 10.3390/biomedicines11061568 37371663 PMC10295799

[8] Zheng S Shi S Ren X Han T Li Y Chen Y. Triglyceride glucose-waist circumference, a novel and effective predictor of diabetes in first-degree relatives of type 2 diabetes patients: cross-sectional and prospective cohort study. J Transl Med (2016) 14:260. doi: 10.1186/s12967-016-1020-8 27604550 PMC5015232

[9] Akinbami LJ Chen TC Davy O Ogden CL Fink S Clark J. National health and nutrition examination survey, 2017-march 2020 prepandemic file: sample design, estimation, and analytic guidelines. Vital Health Stat (2022) 1:1–36. doi: 10.15620/cdc:115434 35593699

[10] Wu Y Chao J Bao M Zhang N Wang L. Construction of predictive model for osteoporosis related factors among postmenopausal women on the basis of logistic regression and Bayesian network. Prev Med Rep (2023) 35:102378. doi: 10.1016/j.pmedr.2023.102378 37662871 PMC10472296

[11] Ruan Z Lu T Chen Y Yuan M Yu H Liu R. Association between psoriasis and nonalcoholic fatty liver disease among outpatient US adults. JAMA Dermatol (2022) 158:745–53. doi: 10.1001/jamadermatol.2022.1609 PMC9134040 35612851

[12] Chen Y Pan Z Shen J Wu Y Fang L Xu S. Associations of exposure to blood and urinary heavy metal mixtures with psoriasis risk among U.S. adults: A cross-sectional study. Sci Total Environ (2023) 887:164133. doi: 10.1016/j.scitotenv.2023.164133 37172860

[13] Liu XC He GD Lo K Huang YQ Feng YQ. The triglyceride-glucose index, an insulin resistance marker, was non-linear associated with all-cause and cardiovascular mortality in the general population. Front Cardiovasc Med (2020) 7:628109. doi: 10.3389/fcvm.2020.628109 33521071 PMC7840600

[14] Bala C Gheorghe-Fronea O Pop D Pop C Caloian B Comsa H. The association between six surrogate insulin resistance indexes and hypertension: A population-based study. Metab Syndr Relat Disord (2019) 17:328–33. doi: 10.1089/met.2018.0122 31034338

[15] Rahmatnezhad L Moghaddam-Banaem L Behroozi-Lak T Shiva A Rasouli J. Association of insulin resistance with polycystic ovary syndrome phenotypes and patients’ characteristics: a cross-sectional study in Iran. Reprod Biol Endocrinol (2023) 21:113. doi: 10.1186/s12958-023-01160-z 38001527 PMC10675950

[16] Sumin AN Bezdenezhnykh NA Bezdenezhnykh AV Osokina AV Kuzmina AA Sinitskaya AV. The role of insulin resistance in the development of complications after coronary artery bypass grafting in patients with coronary artery disease. Biomedicines (2023) 11(11):2977. doi: 10.3390/biomedicines11112977 38001977 PMC10669372

[17] Blount BC Pirkle JL Osterloh JD Valentin-Blasini L Caldwell KL. Urinary perchlorate and thyroid hormone levels in adolescent and adult men and women living in the United States. Environ Health Perspect (2006) 114:1865–71. doi: 10.1289/ehp.9466 PMC1764147 17185277

[18] Kim S Kim S Won S Choi K. Considering common sources of exposure in association studies - Urinary benzophenone-3 and DEHP metabolites are associated with altered thyroid hormone balance in the NHANES 2007-2008. Environ Int (2017) 107:25–32. doi: 10.1016/j.envint.2017.06.013 28651165

[19] Zhang Y Dong T Hu W Wang X Xu B Lin Z. Association between exposure to a mixture of phenols, pesticides, and phthalates and obesity: Comparison of three statistical models. Environ Int (2019) 123:325–36. doi: 10.1016/j.envint.2018.11.076 30557812

[20] O’Hagan R Gonzalez-Cantero A Patel N Hong CG Berg AR Li H. Association of the triglyceride glucose index with insulin resistance and subclinical atherosclerosis in psoriasis: An observational cohort study. J Am Acad Dermatol (2023) 88:1131–34. doi: 10.1016/j.jaad.2022.08.027 35995090

[21] You Y Chen Y Fang W Li X Wang R Liu J. The association between sedentary behavior, exercise, and sleep disturbance: A mediation analysis of inflammatory biomarkers. Front Immunol (2022) 13:1080782. doi: 10.3389/fimmu.2022.1080782 36713451 PMC9880546

[22] You Y Chen Y Zhang Q Yan N Ning Y Cao Q. Muscle quality index is associated with trouble sleeping: a cross-sectional population based study. BMC Public Health (2023) 23:489. doi: 10.1186/s12889-023-15411-6 36918831 PMC10012435

[23] Sala C Morignat E Ducrot C Calavas D. Modelling the trend of bovine spongiform encephalopathy prevalence in France: Use of restricted cubic spline regression in age-period-cohort models to estimate the efficiency of control measures. Prev Vet Med (2009) 90:90–101. doi: 10.1016/j.prevetmed.2009.04.001 19414204

[24] Wu JJ Kavanaugh A Lebwohl MG Gniadecki R Merola JF. Psoriasis and metabolic syndrome: implications for the management and treatment of psoriasis. J Eur Acad Dermatol Venereol (2022) 36:797–806. doi: 10.1111/jdv.18044 35238067 PMC9313585

[25] Bell D Jerkins T. In praise of pioglitazone: An economically efficacious therapy for type 2 diabetes and other manifestations of the metabolic syndrome. Diabetes Obes Metab (2023) 25:3093–102. doi: 10.1111/dom.15222 37534526

[26] Palma A Sainaghi PP Amoruso A Fresu LG Avanzi G Pirisi M. Peroxisome proliferator-activated receptor-gamma expression in monocytes/macrophages from rheumatoid arthritis patients: relation to disease activity and therapy efficacy–a pilot study. Rheumatol (Oxford) (2012) 51:1942–52. doi: 10.1093/rheumatology/kes177 22829690

[27] Xie E Ye Z Wu Y Zhao X Li Y Shen N. Association of triglyceride-glucose index with coronary severity and mortality in patients on dialysis with coronary artery disease. Eur J Med Res (2023) 28:437. doi: 10.1186/s40001-023-01410-1 37848993 PMC10580538

[28] Zhang Q Xiao S Jiao X Shen Y. The triglyceride-glucose index is a predictor for cardiovascular and all-cause mortality in CVD patients with diabetes or pre-diabetes: evidence from NHANES 2001-2018. Cardiovasc Diabetol (2023) 22:279. doi: 10.1186/s12933-023-02030-z 37848879 PMC10583314

[29] Xia W Cai Y Zhang S Wu S. Association between different insulin resistance surrogates and infertility in reproductive-aged females. BMC Public Health (2023) 23:1985. doi: 10.1186/s12889-023-16813-2 37828472 PMC10568938

[30] Al AN Haoudi EN Bensmail H Arredouani A. The triglyceride glucose-waist-to-height ratio outperforms obesity and other triglyceride-related parameters in detecting prediabetes in normal-weight Qatari adults: A cross-sectional study. Front Public Health (2023) 11:1086771. doi: 10.3389/fpubh.2023.1086771 37089491 PMC10117653

[31] Chen L He L Zheng W Liu Q Ren Y Kong W. High triglyceride glucose-body mass index correlates with prehypertension and hypertension in east Asian populations: A population-based retrospective study. Front Cardiovasc Med (2023) 10:1139842. doi: 10.3389/fcvm.2023.1139842 37180805 PMC10166815

[32] Peng H Pan L Ran S Wang M Huang S Zhao M. Prediction of MAFLD and NAFLD using different screening indexes: A cross-sectional study in U.S. adults. Front Endocrinol (Lausanne) (2023) 14:1083032. doi: 10.3389/fendo.2023.1083032 36742412 PMC9892768

[33] Lee HJ Han KD Park HE Han JH Bang CH Park YM. Changes in metabolic syndrome and risk of psoriasis: a nationwide population-based study. Sci Rep (2021) 11:24043. doi: 10.1038/s41598-021-03174-2 34912000 PMC8674228

[34] Liu L Wang W Si Y Li X. Genetic insights into the risk of metabolic syndrome and its components on psoriasis: A bidirectional Mendelian randomization. J Dermatol (2023) 50(11):1392–400. doi: 10.1111/1346-8138.16910 37528547

[35] Egeberg A Gisondi P Carrascosa JM Warren RB Mrowietz U. The role of the interleukin-23/Th17 pathway in cardiometabolic comorbidity associated with psoriasis. J Eur Acad Dermatol Venereol (2020) 34:1695–706. doi: 10.1111/jdv.16273 PMC7496750 32022950

[36] Frankenberg A Reis AF Gerchman F. Relationships between adiponectin levels, the metabolic syndrome, and type 2 diabetes: a literature review. Arch Endocrinol Metab (2017) 61:614–22. doi: 10.1590/2359-3997000000316 PMC10522055 29412387

[37] Gerdes S Rostami-Yazdi M Mrowietz U. Adipokines and psoriasis. Exp Dermatol (2011) 20:81–7. doi: 10.1111/j.1600-0625.2010 21255085

[38] Eirís N González-Lara L Santos-Juanes J Queiro R Coto E Coto-Segura P. Genetic variation at IL12B, IL23R and IL23A is associated with psoriasis severity, psoriatic arthritis and type 2 diabetes mellitus. J Dermatol Sci (2014) 75:167–72. doi: 10.1016/j.jdermsci.2014.05.010 24957500

[39] Lu Y Chen H Nikamo P Qi LH Helms C Seielstad M. Association of cardiovascular and metabolic disease genes with psoriasis. J Invest Dermatol (2013) 133:836–39. doi: 10.1038/jid.2012.366 PMC3570714 23190900

