# Supplementary material for: Positive association between different triglyceride glucose index-related indicators and psoriasis: evidence from NHANES
Source: Front Immunol. 2023 Dec 20;14:1325557. doi: 10.3389/fimmu.2023.1325557 (PMC10765499; doi:10.3389/fimmu.2023.1325557)
Supplement: Supplementary file 1 [file Table_1.docx]

| Exposures | Model 1  Crude OR (95% CI) | Model 2  Adjusted OR (95% CI) | Model 3  Adjusted OR (95% CI) |
| --- | --- | --- | --- |
| TyG-BMI |  |  |  |
| Q1 | Ref. | Ref. | Ref. |
| Q2 | 1.432 (0.843-2.559) | 1.427 (0.836-2.559) | 1.472 (0.858-2.652) |
| Q3 | 2.118 (1.280-3.715) | 2.085 (1.256-3.666) | 2.062 (1.216-3.685) |
| TyG-WC |  |  |  |
| Q1 | Ref. | Ref. | Ref. |
| Q2 | 1.330 (0.790-2.344) | 1.339 (0.789-2.378) | 1.353 (0.793-2.412) |
| Q3 | 1.892 (1.154-3.273) | 1.923 (1.155-3.369) | 1.792 (1.051-3.199) |
| TyG-WHtR |  |  |  |
| Q1 | Ref. | Ref. | Ref. |
| Q2 | 1.727 (1.000-3.173) | 1.689 (0.972-3.122) | 1.716 (0.981-3.185) |
| Q3 | 2.129 (1.254-3.867) | 2.011 (1.169-3.690) | 1.964 (1.115-3.671) |

Table S1. Multivariate regression analysis of TyG-related indicators with psoriasis in supplementary analysis.

Model 1 adjust for: None; Model 2 adjust for: age, sex; Model 3 adjust for: age, gender, education, smoking, drinking, hypertension, diabetes, race/ ethnicity, and glucocorticoids usage; Abbreviation: OR, odds ratios; 95% CI, 95% confidence intervals; TyG-BMI, triglyceride glucose-body mass index; TyG-WC, triglyceride glucose-waist circumference; TyG-WHtR, triglyceride glucose-waist to height ratio; Ref., reference.

Table S2. Multivariate regression analysis of TyG-related indicators, HOMA-IR and QUICKI with psoriasis in supplementary analysis.

| Exposures | Model 1  Crude OR (95% CI) | Model 2  Adjusted OR (95% CI) | Model 3  Adjusted OR (95% CI) |
| --- | --- | --- | --- |
| TyG-BMI | 2.747 (1.708-4.406) | 2.625 (1.621-4.236) | 2.572 (1.532-4.309) |
| TyG-WC | 4.003 (2.182-7.354) | 3.737 (1.994-6.990) | 3.255 (1.644-6.448) |
| TyG-WHtR | 4.239 (2.312-7.786) | 3.747 (1.994-7.032) | 3.720 (1.870-7.408) |
| HOMA-IR | 1.242 (1.076-1.431) | 1.217 (1.052-1.405) | 1.233 (1.052-1.443) |
| QUICKI | 0.221 (0.079-0.608) | 0.256 (0.091-0.713) | 0.234 (0.076-0.708) |

Model 1 adjust for: None; Model 2 adjust for: age, sex; Model 3 adjust for: age, gender, education, smoking, drinking, hypertension, diabetes, and race/ ethnicity; Abbreviation: OR, odds ratios; 95% CI, 95% confidence intervals; TyG-BMI, triglyceride glucose-body mass index; TyG-WC, triglyceride glucose-waist circumference; TyG-WHtR, triglyceride glucose-waist to height ratio; HOMA-IR, the homeostasis model assessment of insulin resistance; QUICKI, the Quantitative Insulin Sensitivity Check Index.
